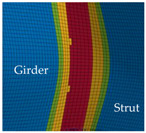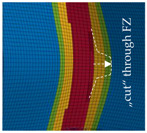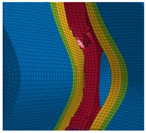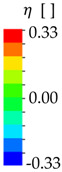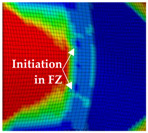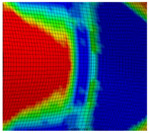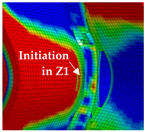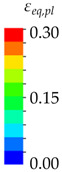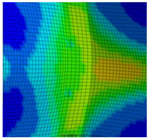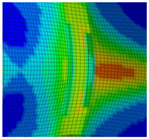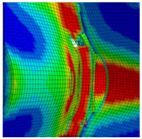# Experimental and Numerical Investigation of the Fracture Behavior of Welded Aluminum Cross Joints under Axial Compression

**DOI:** 10.3390/ma13194310

**Published:** 2020-09-27

**Authors:** Hannes Panwitt, Horst Heyer, Manuela Sander

**Affiliations:** Institute of Structural Mechanics, Faculty of Mechanical Engineering and Marine Technology, University of Rostock, Albert Einstein-Str. 2, 18059 Rostock, Germany; horst.heyer@uni-rostock.de (H.H.); manuela.sander@uni-rostock.de (M.S.)

**Keywords:** welded aluminum alloy, axial compression test, fracture criteria, finite element method

## Abstract

In age-hardened high-strength aluminum alloys, the area with and around a joint has a large impact on the load-bearing capacity of a welded structure. Therefore, in this study the fracture behavior of welded EN AW 6082 T6 plates is investigated experimentally and numerically. From butt joints, smooth and notched tensile specimens as well as shear specimens have been manufactured and tested for the base material (BM), heat-affected zone (HAZ) and fusion zone (FZ). With numerical simulations of these tests, the dependency of the fracture strain on the stress triaxiality is determined, and two phenomenological fracture criteria are calibrated. Whereas the one-parameter Rice–Tracey/Cockcroft–Latham (RTCL) criterion describes the behavior of the tension specimens as accurately as the two-parameter Bao–Wierzbicki (BW) criterion, the BW criterion is more accurate for shear tests. Subsequently, the material model is validated on axial compression tests of welded X-profiles. The experiments comprise tests with different plate thicknesses (8 mm, 10 mm and 12 mm) and varying strain rates (up to 1/s locally), showing the same behavior for all specimens. After crack initiation within the FZ, coalescence of cracks leads to crack growth in axial direction and a subsequent reduction of the load-bearing capacity. This behavior is reproduced well by the numerical simulations with the BW criterion, whereas simulations with the RTCL criterion predict fracture initiation at too high displacements. Overall, the results show the strong influence of the ductility of the FZ on the crushing behavior of welded X-profiles.

## 1. Introduction

High-strength aluminum alloys are the most important lightweight construction materials within the transport industry. They are used not only for aircraft and automobiles but increasingly also for ship constructions. The main hardening process in these alloys is precipitation hardening achieved with natural or artificial ageing. Welding these alloys can result in a loss of strength within the heat-affected zone (HAZ) due to a loss of the ageing state. Furthermore, in contrast to commonly used steel in ship constructions, the fusion zone (FZ) itself can be weaker than the base material of the joined plates. Thus, in case of accidents such as grounding and crashes, the energy absorption of the structure is strongly affected by the welded joints.

On the one hand, the limit load behavior of welded aluminum specimens was investigated numerically and experimentally by several authors for ship constructions [1,2,3,4,5,6,7]. In these studies, the main focus was the global limit load behavior of large structures. Thus, simplifications especially for the fracture behavior had to be made for the HAZ and FZ. On the other hand, there are several approaches to describe in detail the mechanical behavior within the HAZ on small specimens. Several authors performed welding simulations to obtain the properties of the HAZ [8,9,10] or conducted an array of micro-tension tests on specimens manufactured directly out of a HAZ parallel to the weld line [11,12]. Recently, approaches have been proposed for a reduction of the experimental and numerical effort to obtain at least the flow behavior within the HAZ. In these studies, the local strain evolution using strain gauges or digital image correlation (DIC) is evaluated in tensile tests perpendicular to the weld [13,14,15]. In all studies, either the fracture behavior is described in detail without an investigation of the influence on the behavior of larger structures, or simplifications, especially in the HAZ, are made. For example, Urban [1] investigated structural details of welded aluminum X- and T-profiles under axial compression. The experimental results indicate that especially for large plate thicknesses, the folding mechanism is strongly influenced by fracture of the material, thereby reducing the membrane stiffness of the samples and thus drastically decreasing the energy absorption. Whereas with plate thicknesses of 4 mm and 8 mm, fracture occurs within the HAZ, for a plate thickness of 12 mm fracture of the FZ is observed. However, the numerical simulations performed by Urban did not consider the influence of the HAZ and thus lead to under- and overestimation of the energy absorption, depending on the plate thickness. Thus, a proper connection between the local description of the fracture behavior in the area of a weld and the global behavior of ship structures is still difficult, in particular under shear and compression loading.

Therefore, the aim of this study is to investigate the fracture behavior of welded EN AW 6082 T6 plates under various stress states. After a calibration procedure of two phenomenological fracture criteria, numerical simulations of axial compression tests on representative structural details in the form of welded X-profiles are performed and compared with experimental results. To investigate the influence of plate thickness and loading velocity on the fracture behavior of the cross joints, specimens with plate thicknesses of 8 mm, 10 mm and 12 mm are tested with local strain rates up to 1/s for the 8-mm-thick plates.

## 2. Material Characterization

### 2.1. True Stress–Strain Behavior

**Figure 1:**
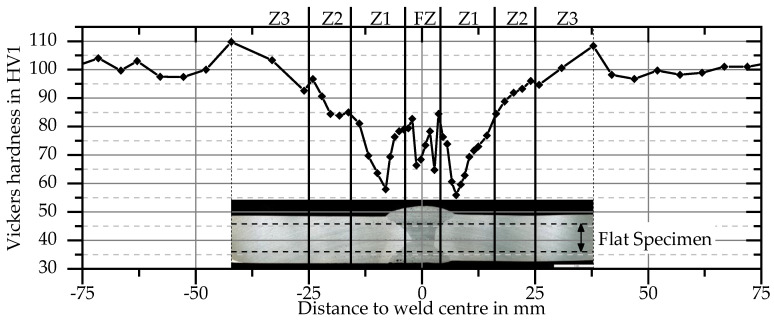
Hardness test after welding and natural ageing in the plate center [16].

**Figure 2:**
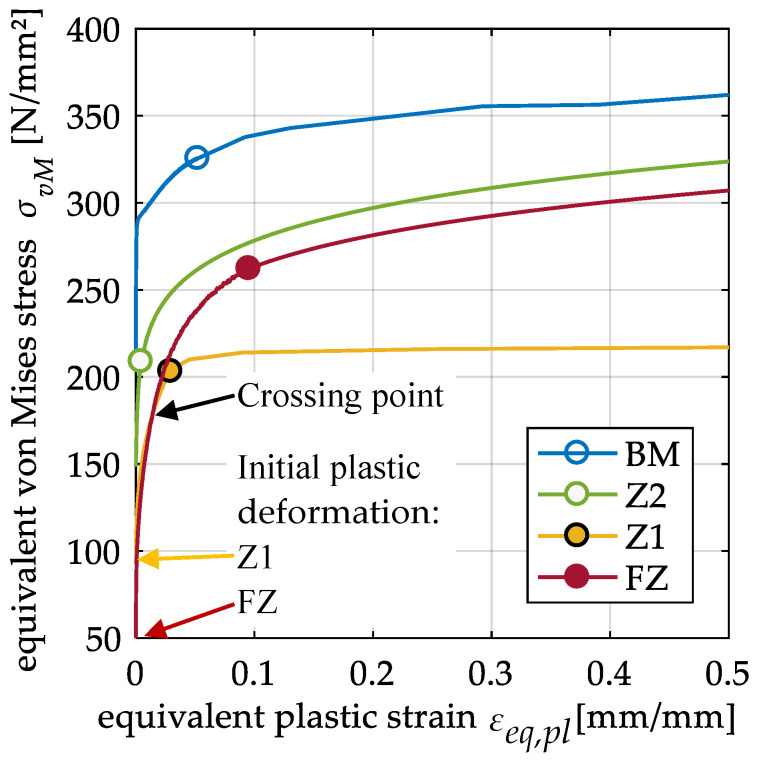
Flow curves of base material (BM), fusion zone (FZ), and heat-affected zone (HAZ) (zone 1 (Z1) and zone 2 (Z2)). The circles mark the last data point directly calculated from measured force–displacement curves (data from [16]).

The material used in this study is a peak hardened aluminum alloy EN AW 6082 T651. This high strength alloy has good weldability, but exhibits a significant reduction in its mechanical properties in the heat affected zone compared to 5xxx-series alloys. In addition, the alloy exhibits little strain rate sensitivity at elevated strain rates. All weldments were manufactured with EN AW 4047 as filler material. The mechanical and thermomechanical behavior of the base material and welded butt specimens was investigated by Wiechmann et al. [16]. A subdivision of the area with and around the weld into four zones delivered accurate results for global force–displacement curves in numerical simulations. These zones consist of the fusion zone (FZ) and three zones in the heat-affected zone (HAZ) and were derived from hardness tests (Figure 1). Because the difference in strength to the base material (BM) is small in zone 3 (Z3), its mechanical properties are considered equal to those of the base material. Thus, for the HAZ in this study only zone 1 (Z1) with the lowest strength and zone 2 (Z2) with an intermediate strength between Z1 and the BM are considered. For these four zones, flow curves were determined from tensile tests with a combined analytical and numerical iterative process. From experimental force–displacement curves the true stress–strain curves were calculated up to the onset of necking. Beyond the onset of necking, the curves of Z1 and the BM have been obtained by iterative numerical simulations. Because the FZ failed without necking and neither necking nor failure occurs in Z2, the experimentally obtained curves were extended with a power law in these two zones. As can be seen in Figure 2, the base material has the highest strength. Furthermore, contrary to the hardness tests, the FZ starts to yield at a lower equivalent stress compared to Z1. However, due to more strain hardening in the FZ, Z1 has the lowest flow stress after exhibiting more than 1.4% plastic strain. The detailed description of the methods and results as well as a detailed investigation on the precipitation behavior of the base material is found in [16].

### 2.2. Phenomenological Damage Model

It is a well-known fact that fracture of ductile metals is strongly dependent on hydrostatic stress. Besides the intensity of the plastic strain, the stress triaxiality

(1) $$\eta =\frac{\sigma_{m}}{\sigma_{vM}}$$

with the hydrostatic stress $\sigma_{m}$ and the equivalent von Mises stress $\sigma_{vM}$ is the most important parameter to describe the initiation of ductile fracture. Depending on the amount of triaxiality, different fracture mechanisms occur. To investigate and characterize the fracture behavior, tests at different stress states are necessary, such as those described by Bao and Wierzbicki [17].

By means of numerical simulations of these experiments, a limiting fracture curve can be determined, which can be described analytically. Based on this, a ductile damage

(2) $$D=\int f\left(\eta \right)d\varepsilon_{eq,pl}$$

can be defined as an integral over plastic strain increments $d\varepsilon_{eq,pl}$ weighted by a damage function f(η). This damage is calculated directly by the implemented damage models of the finite element (FE) solver used in this study, wherein the onset of local fracture processes is characterized by the damage reaching the value one. Thus, within Equation (2) the damage function f(η) can be represented by the inverse of the limiting fracture curve to normalize the plastic strain increments.

For the description of the limiting fracture curve and subsequently f(η), two known analytical functions are used in this study, whose parameters have to be fitted by experiments. The first one is a combination of the criteria by Rice and Tracey [18] and Cockcroft and Latham [19]:

(3) $$f_{RTCL}\left(\eta \right)=\{\begin{array}{ccc} 0 & for & \eta \leq -1/3\\ \frac{2}{\varepsilon_{f,ut}}\frac{1+\eta \sqrt{12-27\eta^{2}}}{3\eta +\sqrt{12-27\eta^{2}}} & for & -1/3<\eta <1/3\\ \frac{1}{\varepsilon_{f,ut}}\frac{\exp\left(\frac{3}{2}\eta \right)}{1.65} & for & \eta \geq 1/3 \end{array}$$

It was proposed as the Rice–Tracey/Cockcroft–Latham (RTCL) criterion somewhat simultaneously by Urban [1] and Törnqvist [2] and requires the fracture strain $\varepsilon_{f,ut}$ in constant uniaxial tension as the only parameter to be calibrated.

The second criterion, the Bao–Wierzbicki (BW) criterion presented by Lee [20], describes the limiting fracture strain curve with a function dependent on two parameters, where one parameter dominates under tension and the second parameter dominates under shear loading. To obtain the damage function f(η), the originally proposed limit curve function is inverted and thus reads as

(4) $$f_{BW}\left(\eta \right)=\{\begin{array}{ccc} 0 & for & \eta \leq -1/3\\ \frac{1+3\eta }{\varepsilon_{f,s}} & for & -1/3<\eta \leq 0\\ \frac{1}{9\left(\varepsilon_{f,ut}-\varepsilon_{f,s}\right)\eta^{2}+\varepsilon_{f,s}} & for & 0<\eta <1/3\\ \frac{3\eta }{\varepsilon_{f,ut}} & for & \eta \geq 1/3 \end{array}$$

where $\varepsilon_{f,s}$ is the fracture strain under constant pure shear (η = 0). For multiaxial compression (η≤−1/3), no damage or fracture of the material is expected [21], and therefore f(η) = 0 in these stress states in both criteria.

For both criteria, the parameter $\varepsilon_{f,ut}$ can be calibrated directly from tensile tests with smooth or notched specimens (η≥1/3) using Equation (2). At fracture initiation (*D* = 1), the uniaxial fracture strain for the RTCL criterion is calculated with

(5) $$\varepsilon_{f,ut}=\int \frac{\exp\left(\frac{3}{2}\eta \right)}{1.65}d\varepsilon_{eq,pl}$$

and for the BW criterion it is calculated with

(6) $$\varepsilon_{f,ut}=\int 3\eta d\varepsilon_{eq,pl}$$

according to which the stress triaxiality during the entire deformation does not have to be constant. Similarly, the second parameter of the BW criterion can be calibrated with

(7) $$\varepsilon_{f,s}=\int \left(1+3\eta \right)d\varepsilon_{eq,pl}$$

using a shear test (−1/3<η≤0). When a test is conducted, where the stress triaxiality is in the range of 0<η<1/3, iterative numerical simulations have to be performed to calibrate the parameter $\varepsilon_{f,s}$.

### 2.3. Experimental Investigations

**Figure 3:**
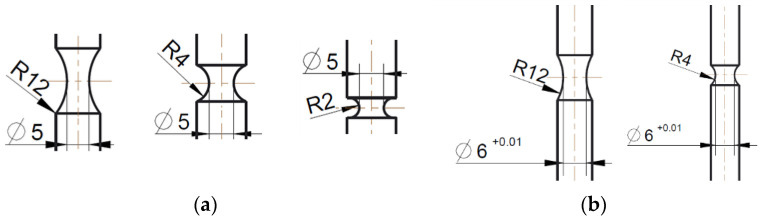
Notched round tensile specimens of (**a**) the BM and (**b**) the FZ.

Combined experimental and numerical investigations are used to determine the fracture strains according to Equation (5) or (6) for the four defined zones in the region of a weld seam. Tensile tests were carried out on smooth round specimens to determine the fracture strain in the BM and the FZ. For the validation of the fracture criteria under high stress triaxialities, three different notched specimens of the BM (Figure 3a) and two different specimens of the FZ (Figure 3b) were manufactured and tested.

With these geometries, initial stress triaxialities of 1/3≤η≤0.84 for the BM and 1/3≤η≤0.56 for the FZ were realized. All round specimens were tested in a 50-kN servo-hydraulic uniaxial tension testing machine (Instron GmbH, Darmstadt, Germany), from which the cylinder forces were recorded. Extensometers (MTS 634.31F-24, from MTS Systems GmbH, Berlin, Germany) with a gauge length of 39.4 mm and 30.0 mm were used to measure the displacements on the specimens’ surface of the BM and FZ specimens, respectively. For all geometries, a set of three specimens were tested.

Welded flat specimens with notches of different radii in Z1 (described in detail by Wiechmann et al. [16]) were used to determine the fracture strains in the HAZ. The welded specimens were tested in a 100-kN servo-hydraulic uniaxial tension testing machine (MTS). A digital image correlation system was installed to measure the displacements on the specimens’ surfaces. With these tests, initial stress triaxialities of 1/3≤η≤0.52 were realized.

**Figure 4:**
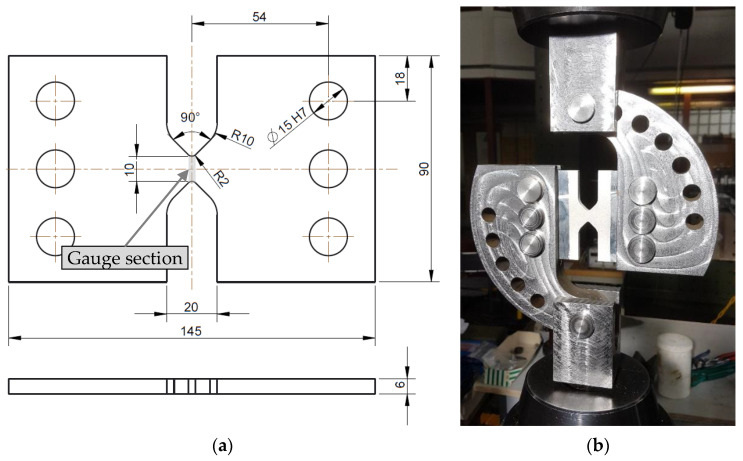
(**a**) Geometry of the shear specimen; (**b**) shear specimen mounted in the corresponding loading device for testing in a uniaxial testing machine.

**Figure 5:**
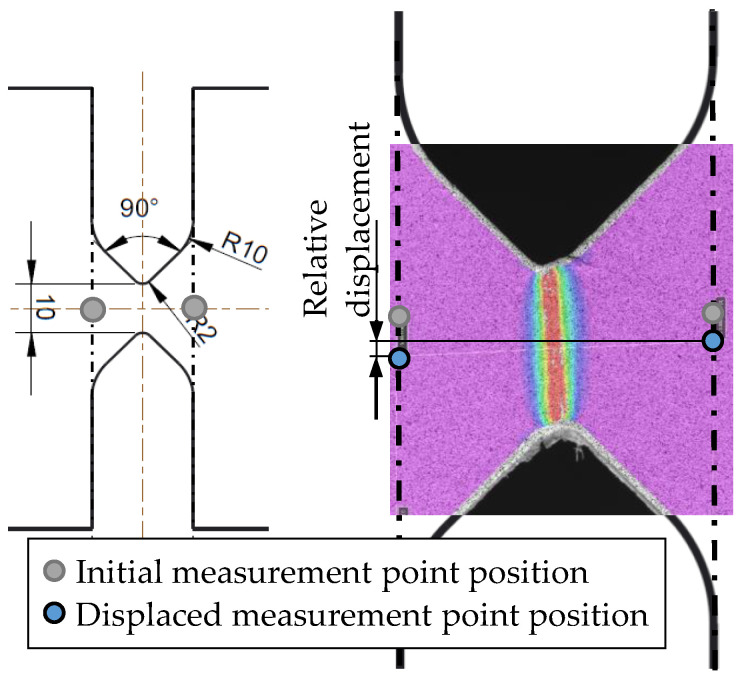
Measurement of the relative displacement in a shear test with digital image correlation (DIC).

To determine the fracture strain for pure shear (η=0) according to Equation (7), a shear specimen was developed for this study (Figure 4a). This specimen is based on the fracture mechanical compact tension shear specimen developed by Richard [22] and needs less weld length per specimen than a specimen according to the standard ASTM B0831. The specimen is tested with the corresponding loading device in the 100-kN servo-hydraulic uniaxial tension testing machine (Figure 4b). The shear specimens were manufactured from the base material and from welded butt welds with the same welding parameters as described by Wiechmann et al. [16]. The respective gauge section was placed in the middle of the FZ or in the HAZ at a distance of 10 mm from the center of the FZ depending on the fracture strain to be determined (Figure 4a). All specimens were milled to a thickness of 6 mm to eliminate welding deformations. Furthermore, by removing the excess FZ material, fracture failure in the gauge section is ensured when testing the FZ. All shear specimens were prepared for two-dimensional digital image correlation (DIC) to measure the displacements and determine strains on the specimens’ surface in the post-processing. Therefore, one side of the specimens was painted with a speckle pattern of black airbrush dots on a white base coat. The 12 MP (4096 × 3000 pixels) camera recorded the tests with a resolution of approximately 133 pixels/mm. With this setup, the positions of subsets (subset area: 29 × 29 pixels) with 7-pixel distance to each other were tracked in post-processing with the software Vic-2D 6 (Correlated Solutions, Irmo, SC, USA). To determine the global structural behavior of the shear test specimens, the relative displacements in the loading direction of two defined points are measured in addition to the forces (Figure 5). Furthermore, in corresponding FE simulations, node displacements at the same positions on the specimens’ surfaces can be evaluated to compare the global responses.

### 2.4. Results and Calibration of Fracture Criteria

Force–displacement curves have been obtained from all individual experiments and averaged for each specimen geometry. To obtain the necessary information on plastic strain and stress triaxiality evolution during the tests, FE analyses of all specimens have been performed. For the round specimens, four-node axisymmetric elements have been used to discretize half models with the symmetric plane perpendicular to the axis of revolution. The numerical simulations on welded flat specimens have been performed with solid hexahedral elements in quarter models and have been evaluated by Wiechmann et al. [16]. All shear specimens were also modeled with three-dimensional solid hexahedral elements. Here, only symmetry in the thickness direction could be utilized. For comparability in the simulations, displacements were obtained at nodes at the same position as the real or virtual extensometers, i.e., on the specimens’ surface. All simulations were performed with MarcMentat2013 and LS-DYNA (v10.1 SMP). Similar element sizes were used in all simulations to minimize the influence of the element size on the local results.

**Figure 6:**
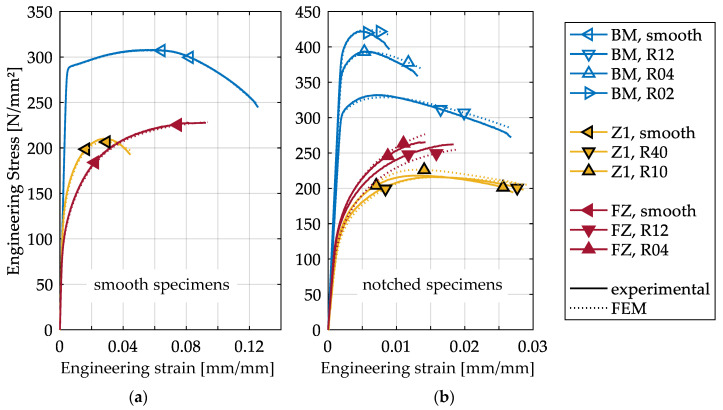
Comparison of experimentally and numerically determined engineering stress–strain curves of smooth (**a**) and notched (**b**) tensile specimens of the BM, FZ and HAZ. The experimental results are averaged results from series of three tests. The numerical results are displayed up to the experimentally determined fracture displacement.

For the tensile tests, engineering stress–strain curves have been calculated from the corresponding force–displacement curves to compare the global responses of the specimens (Figure 6). The results not only show that the Z1 has the lowest strength but also that Z1 is less influenced by decreasing the notch radius than the FZ and BM. This shows the strong influence of the inhomogeneous material on the local stress state in the HAZ [16], whereas in the FZ and the BM, the specimen geometry is dominant. Furthermore, Figure 6 shows that the numerical simulations are in good agreement with the experimental results. The numerical simulations indicate that crack initiation starts in the interior of the specimens in all tests, where the highest stress triaxiality and strain localization occur at the experimental fracture displacement. Thus, the evolution of triaxiality and strains in the center of the specimens are used for the calibration of the fracture criteria.

**Figure 7:**
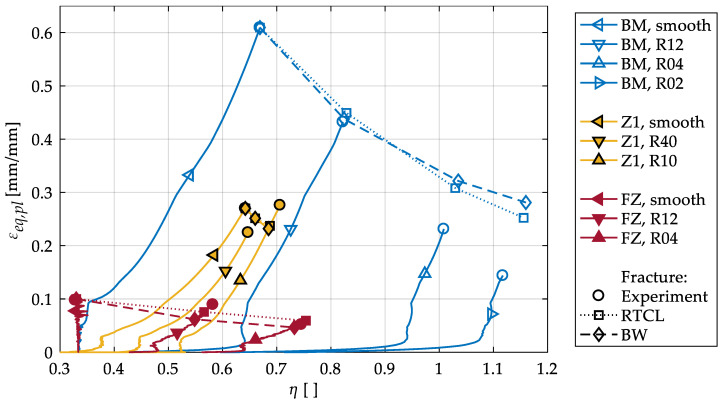
Calibration of $\varepsilon_{f,ut}$ of the Bao–Wierzbicki (BW) and Rice–Tracey/Cockcroft–Latham (RTCL) criteria with the evolution of $\varepsilon_{eq,pl}$ over η in smooth and notched tensile tests for BM, HAZ and FZ.

As can be seen in Figure 7, the stress triaxiality is not necessarily constant during a tensile test, even for a smooth specimen. The triaxiality remains constant only in smooth specimens of the FZ, because brittle fracture leads to failure without necking. In tests on smooth BM specimens, the triaxiality increases immediately after necking occurs, in the present results up to η = 0.67. In Z1, no constant triaxiality can be observed, even in smooth specimens, because the material inhomogeneity causes an immediate increase in the triaxiality under tension. An immediate increase in stress triaxiality is also visible for all notched specimens because the geometry hinders a homogeneous deformation.

Furthermore, Figure 7 shows the plastic strain and triaxiality at the experimental fracture displacement. For the BM and FZ, a monotonically decreasing fracture strain is visible for decreasing notch radii (i.e., increasing η), which is in accordance with results in the literature for homogeneous aluminum alloys [17,23,24,25]. However, for the HAZ the highest fracture strain is observed for the smallest notch radius. To the authors’ knowledge, there are no comparable experiments on the HAZ in the literature. It has to be considered that the subdivision of the HAZ into two zones leads to an averaging of the mechanical properties within Z1, which results in an increase in the lowest yield strength. This may not only cause the maximum local strains to be lower than in the experiments [16] but may also cause the observed increase in the simulated fracture strain at the lowest notch radius. An influence of the manufacturing process on the results might also be a reason, because the specimens with a 10-mm notch radius were manufactured from a different half of the same welded plate as the 40-mm notch radius specimens. As the heat flow generated by welding at the end of a weld seam can differ from the beginning of the seam, different ductilities at a 10 mm distance to the center of the FZ might be the result. However, the displacements, triaxialities and plastic strains at fracture within the various HAZ specimens are very similar, confirming the strong influence of the material inhomogeneity on the $\varepsilon_{eq,pl}$–η evolution compared to the influence of the geometry.

To calibrate the fracture criteria, the parameter $\varepsilon_{f,ut}$ has been calculated from the $\varepsilon_{eq,pl}$–η evolution in the smooth specimens for the RTCL and BW criteria with Equations (5) and (6), respectively. The calculated values are listed in Table 1. The parameters for Z2 are obtained by averaging the values from Z1 and the BM, since an intermediate behavior can be expected from the hardness measurements and thermomechanical analyses conducted by Wiechmann et al. [16].

Figure 7 shows that the resulting crack initiation loci within the $\varepsilon_{eq,pl}$–η evolution is predicted similarly with both criteria for all specimens. The simulated fracture strains are slightly too high at small notch radii for the BM. The behavior of the HAZ (increasing fracture strain with decreasing notch radius) cannot be modeled with these criteria. However, the differences to the experimental fracture strains are small for both notched specimens, because all fracture loci are close to each other.

**Figure 8:**
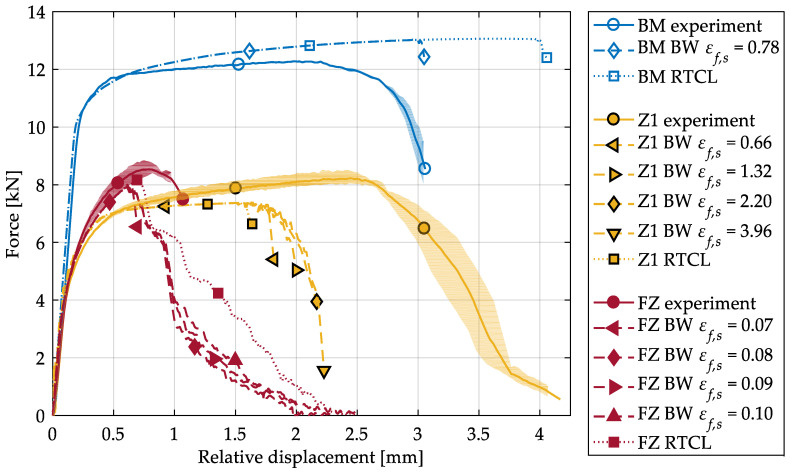
Force–displacement curves of shear tests obtained by experiments and numerical simulations with both fracture criteria (dotted: RTCL and dashed: BW) for three material zones. The experimental results (solid lines) are shown with their respective scatter band. $\varepsilon_{f,ut}$ is the constant value obtained from tensile tests during variation of $\varepsilon_{f,s}$. The simulation with the parameters displayed in Table 1 for the BW criterion is marked with a diamond.

**Figure 9:**
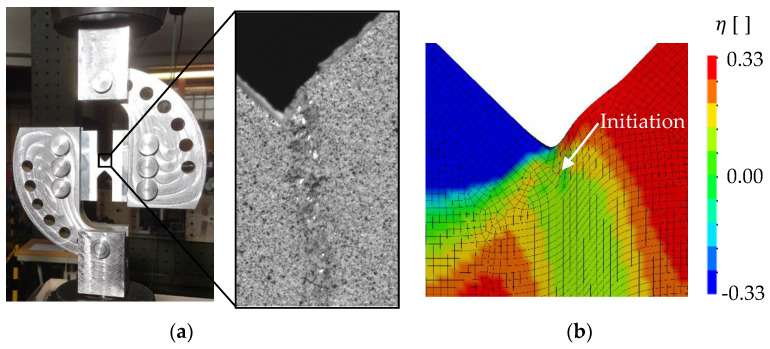
Fracture behavior at the surface of the BM shear specimen in (**a**) the experiment and (**b**) finite element (FE) simulations with the BW criterion.

To calibrate the shear-dominating parameter $\varepsilon_{f,s}$ of the BW criterion, the shear tests have been evaluated and simulated numerically. The experimentally and numerically obtained force–displacement curves are shown in Figure 8. Whereas the RTCL criterion overestimates the experimental fracture displacement for the BM, the prediction with the BW criterion after calibration of $\varepsilon_{f,s}$ is accurate. As Figure 9a shows, a very localized shear band forms in the experiments. Within this shear band, crack initiation (in the sense of a macrocrack) is predicted at the surface by the BW criterion (Figure 9b), resulting in the same fracture path as in the experiments. In the following, crack initiation shall always be understood as local failure, i.e., the formation of a macrocrack. Both models were not able to reproduce the decrease in force, before complete fracture of the ligament occurs, which might be enhanced by shear-induced softening. To model this softening behavior, a coupling of damage and the plastic behavior is required, but this was not considered in the present material model.

**Figure 10:**
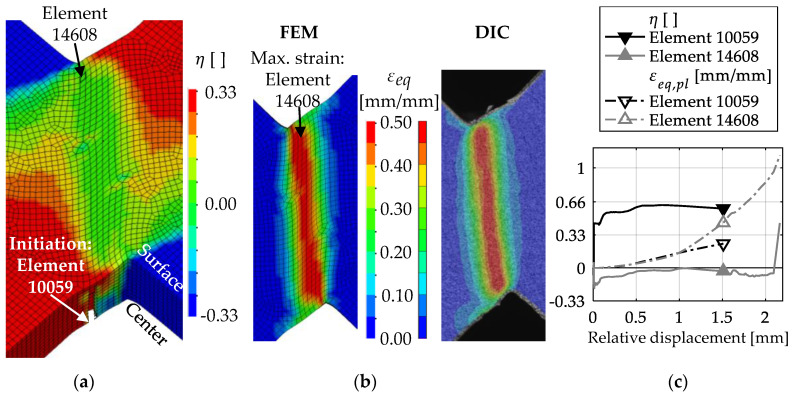
Stress triaxiality and strain within Z1 in shear tests: (**a**) η at 1.7-mm displacement and location of crack initiation with BW criterion; (**b**) comparison of $\varepsilon_{eq}$ on the surface obtained by FE modeling (FEM) and DIC at 1.7-mm displacement; (**c**) evolution of η and $\varepsilon_{eq}$ at the location of crack initiation with the BW criterion (element 10059) and the location of the highest strain (element 14608); the markers are set at the displacement of crack initiation.

As can be seen from Figure 8, the numerical simulations failed to model the global behavior of Z1 in the shear tests. In the simulations, cracks initiate and grow at a stress triaxiality of η > 1/3 in the symmetry plane of the specimens (Figure 10a). However, the highest strain (element 14608) occurs at the specimen’s surface (Figure 10b). Whereas at the location with the highest strain the stress triaxiality stays at the designed η ≈ 0 until complete fracture, crack initiation (element 10059) occurs at a comparatively low strain (Figure 10c). This behavior is caused by the chosen simplification of the HAZ, in particular because of the subdivision of the HAZ in only two zones. In the simulations of the tensile tests, local strains and consequently $\varepsilon_{f,ut}$ are underestimated. In the simulations of the shear tests, this leads to crack initiation and growth in regions with low strain but high stress triaxiality, i.e., at low displacements. As a result, Equation (7) could not be used for determination of $\varepsilon_{f,s}$, since Equation (7) is only valid for η ≤ 0. Modifying $\varepsilon_{f,s}$ in numerical simulations shows that the influence of shear-dominated damage accumulation on the fracture displacement is minor compared to tension-dominated accumulation. Thus, simulations with $\varepsilon_{f,s}\;\geq$ 2.20 do not result in an increase in the fracture displacement of more than 2.25 mm. Therefore, $\varepsilon_{f,s}$ = 2.20 was used as the calibrated parameter for Z1, as this provides the best approximation of the experimental fracture displacement.

**Figure 11:**
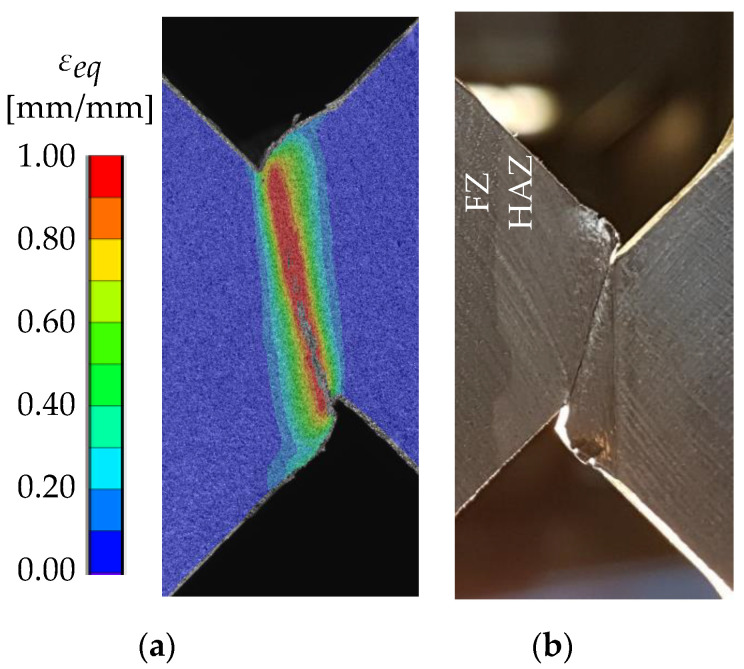
(**a**) Strain distribution at 3.25-mm displacement and (**b**) the backside of the specimen at about 3.5-mm displacement.

Figure 10b shows that the distribution of the equivalent strain $\varepsilon_{eq}$ in the simulations is in good agreement with the experimental results obtained by DIC up to a 1.7-mm displacement. It has to be noted that for the comparison of the local strains in the experiments and simulations, the equivalent strain $\varepsilon_{eq}$ is used instead of $\varepsilon_{eq,pl}$, because DIC results inherently contain both the elastic and plastic strains. However, for the large strains mainly investigated in this study, the difference between $\varepsilon_{eq}$ and $\varepsilon_{eq,pl}$ is small. The DIC results also show that strains of at least $\varepsilon_{eq}$ = 1.0 occur within the localized shear band (Figure 11a). Moreover, it can be assumed that local strains exceed 100% before fracture of the ligament, because the strain distribution shown in Figure 11a occurs at 3.25 mm relative displacement (i.e., 0.75 mm before fracture of the ligament). The narrow band of deformation (Figure 11b) also indicates the increasing localization. However, higher strains could not be measured due to failure of the coating.

With the described method, contrary to the observations by Bao and Wierzbicki [17] for EN AW 2024 T351, the obtained fracture strain at pure shear in Z1 is considerably higher than the fracture strain at uniaxial tension (Table 1). Higher fracture strains under shear loading can be observed for materials such as EN AW 1100 and copper [26]. However, the large difference between $\varepsilon_{f,s}$ and $\varepsilon_{f,ut}$ presented here is assumed to be a consequence of the simplifications within the HAZ.

**Figure 12:**
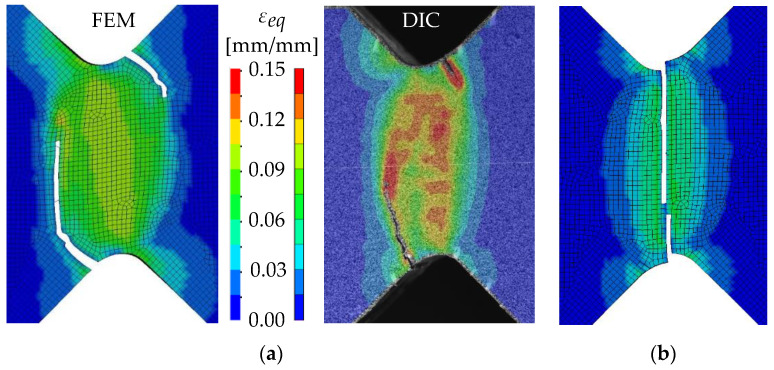
(**a**) Comparison of the fracture behavior of the FZ in shear tests and in numerical simulations with the calibrated BW criterion ($\varepsilon_{f,s}$ = 0.08) and (**b**) fracture behavior simulated with the BW criterion with $\varepsilon_{f,s}$ = 0.07.

Compared to the BM and HAZ, the crack initiation and growth in the FZ specimens are different (Figure 12a right). At first, two cracks initiate on opposite sides of the specimens perpendicular to the direction of the greatest principal normal stress. These cracks grow until one becomes dominant. Crack initiation and fracture of the ligament occur at very low local strains (<20%) compared to the BM and the HAZ specimens. If calibrating the BW criterion on these results, a change of fracture behavior depending on the parameter $\varepsilon_{f,s}$ can be observed. Whereas for $\varepsilon_{f,s}$ < 0.08 the specimen fails due to shear (Figure 12b), for $\varepsilon_{f,s}\geq$ 0.08 the same fracture behavior as in the experiments is simulated (Figure 12a; left). Since in the latter case the crack initiation is caused by tension, increasing $\varepsilon_{f,s}$ has almost no effect on the maximum force. Instead, the fracture of the ligament is mainly affected by $\varepsilon_{f,s}$, resulting in a higher displacement for complete fracture (Figure 8). In any case, after the crack initiation the drop of force is higher than in the experiments, but no fracture of the ligament is observed. Due to the stable crack growth in the simulations, the simulated displacement at complete fracture is higher than in the experiments. The best approximation of the final displacement at fracture was achieved with the calibrated value of $\varepsilon_{f,s}$ = 0.08. In comparison to the calibrated BW criterion, the simulations with the RTCL criterion predict a higher maximum force and a lower drop of force after crack initiation (Figure 8). However, the crack growth behavior is similar to the calibrated BW criterion and thus resembles the crack path in the experiments.

When comparing the fracture strains of the HAZ and BM with the FZ (Table 1 and Figure 7) in a wide range of η, the low overall ductility of the FZ becomes obvious. Microsections of the FZ (see also Section 3.3) reveal pores as a result of the welding process. According to the theory of void growth and coalescence in the ductile damaging process, these pores act as a large initial void volume fraction within the FZ and subsequently lead to the low observed fracture strains. Furthermore, in the shear tests of the HAZ and BM, single localized shear bands are formed, whereas two cracks initiate and grow within the FZ. This shows that the fracture behavior in a wide range of stress triaxialities might not be the same for different aluminum alloys and ageing states, respectively.

## 3. Axial Compression Tests on X-Profiles

### 3.1. Test Setup

**Figure 13:**
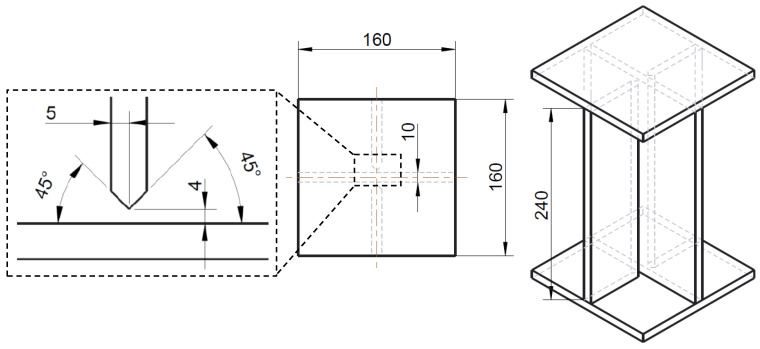
Dimension of an X-profile with a 10-mm plate thickness (without weld seams).

For the investigation of the crushing behavior of welded aluminum structures, X-profiles have been manufactured. As can be seen in Figure 13, additional endplates are welded onto the ends of the cross joint section. This ensures a force transmission directly into the plates’ planes in an axial compression test. The cross joint section (i.e., between the endplates) of the specimens has a height of 240 mm and a width of 160 mm. To manufacture the cross joint, two shorter struts are welded manually with metal inert gas (MIG) welding onto an undivided 160-mm-wide girder. To avoid weld gaps in the root of the T-joints, the welding is performed as double HV- (or K-) welds with at least three welding beads, depending on the plate thickness. For this study, specimens with plate thicknesses of 8 mm, 10 mm and 12 mm are investigated with equivalent preparation of the struts for all thicknesses (Figure 13; left). For all welds, the base material was EN AW 6082 T651 with EN AW 4047 as filler material (wire diameter 1.2 mm). As the shielding gas, a mixture of argon and helium (70%/30%) protects the welding process from air. For the first weld bead of each T-joint, a ceramic weld pool backing was used. In addition, between two welds, the specimens were able to cool down below 100 °C to ensure the same conditions for each weld. After the welding of the cross joint, endplates were attached with simple fillet welds on both sides of each plate.

**Figure 14:**
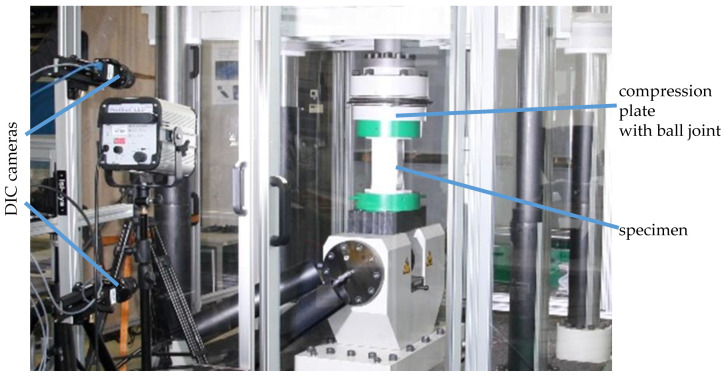
Test setup for the axial compression test with DIC cameras in place.

The X-profiles were tested with a servo-hydraulic press with a maximum capacity of 1300 kN (Figure 14). While the lower compression plate is rigid, the ball joint of the upper compression plate prevents unintended application of moments into the specimens. Lateral forces were minimized due to an accurate alignment of the specimens with the axis of loading. During all tests, the force and displacement of the hydraulic cylinders were recorded by the system. In order to determine the stiffness of the press for the expected ultimate load of the X-profiles and to calibrate the cylinder displacement, the compression plates were compressed against each other up to 1200 kN. Additionally, to obtain displacements and strains from the surface of the specimens, a three-dimensional digital image correlation system was used. Since large buckling is expected to occur to an unknown side of the specimens, two 5 MP cameras (2048 × 2448 pixels) were positioned vertically (Figure 14). In that way, the recording of the deformation of one half of the specimens for the whole experiment is ensured. The working distance was chosen so that the whole specimen was maximized in the pictures. In the area of interest, the resultant resolution was 8.5 pixels/mm on average. The exact determination of the camera positions in space was performed according to a calibration routine recommended by Correlated Solutions. Therefore, pictures of a grid target in different positions and orientations were evaluated by the software Vic3d 7. In the post-processing with Vic3d 7, the positions of subsets (subset area: 39 × 39 pixels) with 7-pixel distance to each other were tracked with this setup to calculate displacements and strains. For a better resolution of the local strains, the tests on the 12-mm specimens were recorded with the same test setup but with two 12 MP cameras (4096 × 3000 pixels), resulting in an average of 12.6 pixels/mm and a subset area of 39 × 39 pixels.

The tests on the specimens with 10-mm and 12-mm plate thicknesses can be considered as quasi-static, since a traverse velocity of 0.01 mm/s was chosen. The traverse velocity of the tests on the specimens with an 8-mm plate thickness was varied between quasi-static (0.01 mm/s) and the maximum possible velocity of 45 mm/s; the velocity was varied with 10 mm/s, 25 mm/s and 40 mm/s in between.

### 3.2. Finite Element Model of the Axial Compression Test

For the numerical simulations of the axial compression tests (10-mm plate thickness), the explicit solver of LS-DYNA was used. The model consists of one-point reduced integration hexahedral elements with at least four elements in the thickness direction of the plates, since simulations with four-node quadrilateral shell elements underestimate the load-bearing capacity of the profile after buckling [27]. To obtain accurate results for the ultimate load of the structure, geometric imperfections have to be considered in the model. Therefore, the surface geometry of four specimens with a 10-mm plate thickness has been measured initially with strip light projection. Systematic numerical simulations considering these imperfections show that bulging of the plates as a result of the welding process has the major influence on the structural behavior [28]. Thus, in the present numerical model only the bulging is considered and described with an idealized sinusoidal imperfection.

(8) $$f\left(x,y\right)=a_{I}sin\left(\frac{\pi }{b}x\right)cos\left(\frac{\pi }{h}\left(y-\frac{h}{2}\right)\right)$$

**Figure 15:**
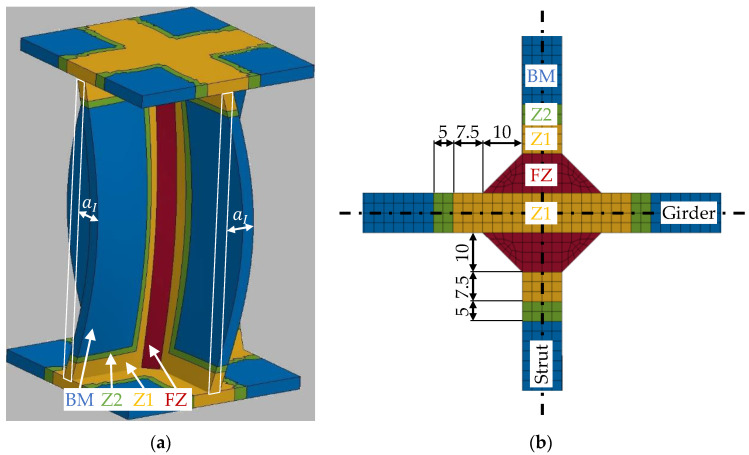
Geometry of the X-profile: (**a**) X-profile with a scaled idealized imperfection; (**b**) cross section in LS-DYNA with dimensions of the material zones.

Each node in the FE model is moved according to Equation (8) perpendicular to the plates’ plane, where b is the width, h is the height of the specimen, and $a_{I}$ is the maximum distance perpendicular to the plane. For this study, $a_{I}$ = 0.1 mm was determined as the result of the measurements in [28] corresponding to 10% of the plate thickness, which is in agreement with similar simulations in the literature [1]. A model with a scaled imperfection is shown in Figure 15a for visualization.

**Figure 16:**
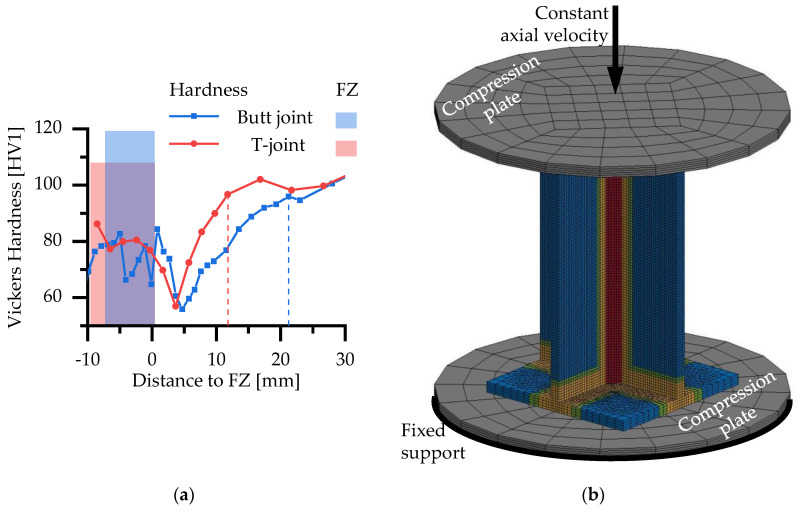
(**a**) Comparison of hardness in a butt weld and T-joint [16]; (**b**) FE model of the axial compression tests with 10-mm plate thickness.

The shape of the fusion zone is idealized to a triangular shape with a design throat thickness of 10 mm (Figure 15b). In [16], it has been shown that the HAZ in a T-joint with similar welding parameters to the present T-joints in the X-profiles is smaller than the HAZ in the investigated butt joints (Figure 16a). The same hardness as in the base material (99 HV) is reached within 12.5 mm distance to the weld seam in the T-joint in comparison to 22 mm in the butt joint. Therefore, the size of Z1 and Z2 in the FE model of the X-profile (Figure 15b) is reduced compared to the models with butt welds (tensile and shear specimens). While the HAZ next to the fillet welds at the endplates of the X-profile is also considered, the fillet welds themselves are not modeled. Instead, the ends of the X-profile are connected directly to the endplates due to shared nodes. The flow curves presented in Section 2.1 were assigned to the respective material zones as piecewise linear curves. In addition, the RTCL and BW criteria (Section 3.4) were implemented in the model for each material zone.

In addition to the specimen itself, two compression plates with linear-elastic material characteristics of steel have been modeled. While the lower one was fixed at the bottom, the upper one was moved with a constant velocity in the axial direction (Figure 16b). A contact condition between the plates and the specimen ensured the transmission of force.

### 3.3. Experimental Results and Discussion

**Figure 17:**
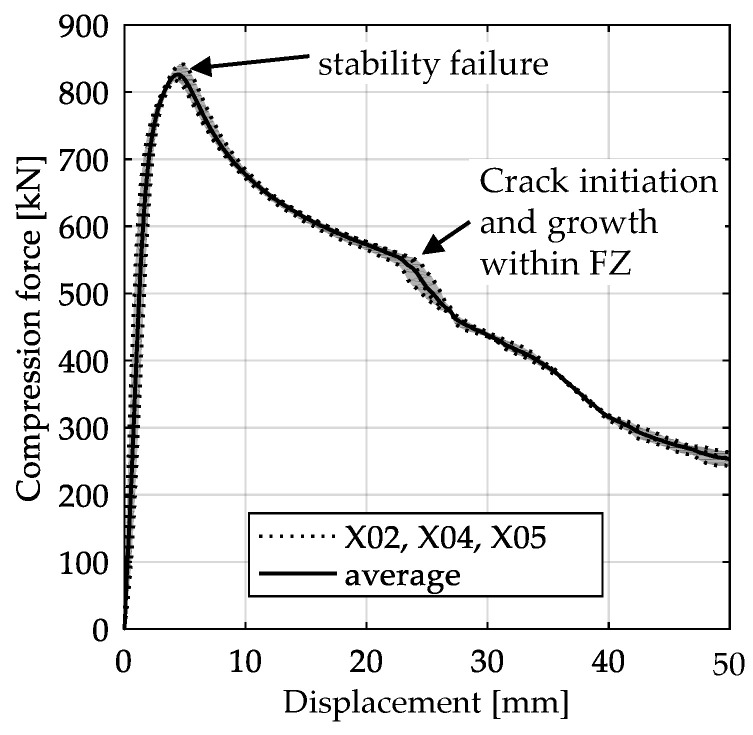
Averaged force–displacement curve with scatter band of axial compression tests with 10-mm plate thickness (data from [27]).

The global response of all axial compression tests is captured by the force–displacement curves [27] and shown in Figure 17 for specimens with a 10-mm plate thickness. All specimens behave quite similarly. Before reaching the limit load, the structures already exhibit plastic deformations. At the displacement related to the maximum force, the structures start to buckle, and therefore stability failure occurs. Table 2 shows the deformation behavior of all specimens at 25-mm and 42-mm displacement. Furthermore, at 25-mm displacement, cracks within the FZ have initiated in the center of the specimens. This crack initiation and growth in the FZ causes the drop of force after a displacement of 22–25 mm visible in Figure 17. With increasing compression, the crack growth progresses within the FZ in the axial direction for all specimens (Table 2, 42-mm row). At displacements of more than 50 mm, self-contact can start to occur, which is not of interest in this study.

**Figure 18:**
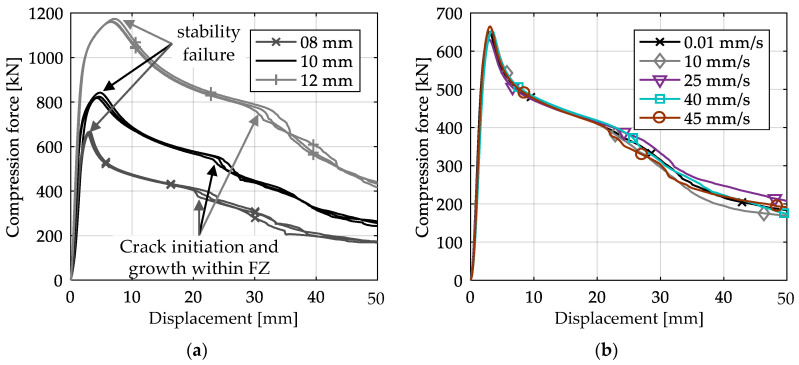
Force–displacement curves of axial compression tests with (**a**) various plate thicknesses and (**b**) various loading velocities for 8-mm plate thickness (averaged curves).

In Figure 18a, the force–displacement curves of the axial compression tests of X-profiles with different plate thicknesses are compared. Whereas the principal response of the specimens is the same for all plate thicknesses, the load-bearing capacity of the structure decreases with decreasing plate thickness. Due to the thinner plates, the maximum force and therefore the stability failure occur at the lowest displacements for the 8-mm plate thickness. With decreasing plate thickness, local failure due to crack initiation can therefore also be determined at lower global displacements.

**Figure 19:**
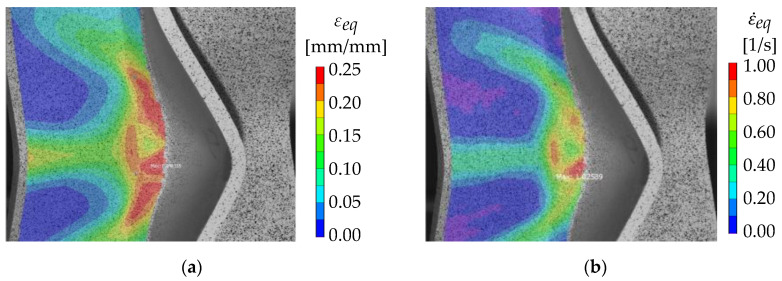
(**a**) Equivalent strain and (**b**) strain rate of a specimen tested at 45 mm/s loading velocity immediately before crack initiation calculated by DIC.

Figure 18b shows the force–displacement curves of X-profiles with 8-mm plate thickness and different loading velocities. The curves are averaged over each series of tests. All curves are very similar until crack initiation in the fusion zone. With increasing displacement, the curves start to vary more, with the highest energy absorption (area below the curve) at 25 mm/s and the lowest energy absorption at 10 mm/s. The curves of the highest tested velocities are almost identical to the quasi-static tests, which is the reference curve here. Additionally, all tests can be considered as inside the same scatter band. For one specimen at the highest loading velocity, the distributions of equivalent strain and equivalent strain rate have been calculated by DIC (Figure 19). As shown in Figure 19b, the maximum strain rate reaches 1/s within the fusion zone before cracking occurs. Thus, from the present results, it can be concluded, that the investigated welded joints have no strain rate dependence under axial compression up to local strain rates of 1/s. These results are in accordance with strain rate tests on welded aluminum specimens [29,30].

**Figure 20:**
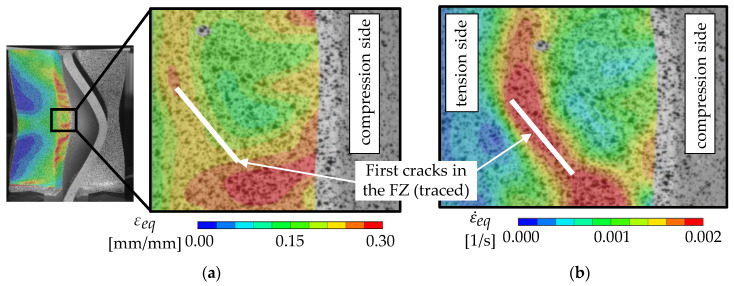
(**a**) Equivalent strain and (**b**) strain rate in the fusion zone of a 12-mm-plate-thickness specimen immediately before crack initiation calculated by DIC.

Further investigations of the strain distribution at crack initiation reveal that crack initiation can occur at locations with less than the maximum equivalent strain (Figure 20a). This shows that the crack initiation is not only dependent on the maximum strain, but it is also influenced by e.g., the stress state and microstructure. Figure 20b shows that the strain rate can have its maximum at the location of crack initiation and not at the location with the highest strain overall, also indicating the enhanced damage accumulation there.

**Figure 21:**
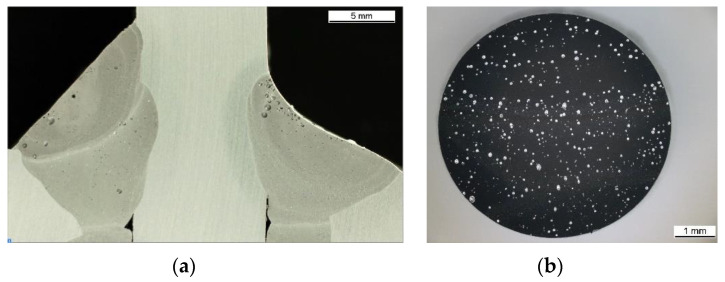
Cross section of (**a**) an X-profile and (**b**) a tensile specimen of the FZ material [34].

Microsections of an X-profile (Figure 21a) and a tension specimen (Figure 21b) reveal that pores are present in the FZ. Under tension, it is widely accepted that void growth and coalescence are the main damaging mechanisms and are enhanced strongly by these pores. However, under shear loading the damage mechanisms are still discussed in the literature, but experimental and numerical results indicate that initiation of micro cracks after elongation and flattening of the pores are the main damage mechanisms in low stress triaxialities [31,32,33]. Thus, the localization of strains (Figure 20a) and strain rate (Figure 20b) at different locations is associated with the formation of microcracks under the surface of the FZ. However, microcracks are not investigated directly in this study. After formation of microcracks, the strain rate can increase drastically, since the material has less resistance. The formation itself does not necessarily have to take place at the location with the highest strain, because the damaging process is strongly dependent on the presence of pores and the stress state. Because the distribution of pores under the surface is assumed to be somewhat homogenous, the location of macrocrack initiation indicates a gradient of the stress state. On the compression side of the buckles, negative stress triaxialities can be expected, and therefore less damage is accumulated than on the tension side. Thus, the critical damage for crack initiation within the material can be reached on the tension side at lower strains than on the compression side and explains the crack initiation behavior shown in Figure 20. Note that these cracks do not necessarily influence the global behavior of the specimen, since cracks of several mm in length are visible at the specimen’s surface well before the compression force drops. This indicates that there needs to be a certain amount of coalescence of cracks, before the global behavior is affected.

### 3.4. Numerical Simulation of the Compression Tests

**Figure 22:**
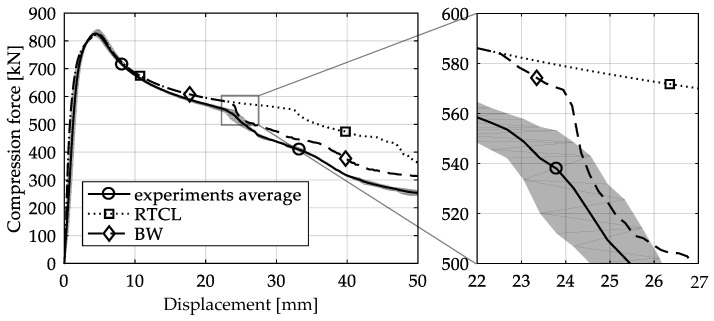
Force–displacement curves of numerical simulated axial compression tests in comparison with the experimental average and its scatter band. The curves’ progressions at crack initiation is shown in detail on the right.

The global results of the numerical simulations of the axial compression tests are shown in Figure 22 in the form of force–displacement curves. The simulations with an imperfect geometry (see Section 3.2) predict the instability failure and the drop of force until crack initiation in the FZ in accordance with the experiments. Whereas the load-bearing capacity after 25-mm displacement is strongly overestimated in simulations with the RTCL criterion, simulations with the BW criterion lead to a smaller overestimation.

The detail in Figure 22 shows that the BW criterion predicts the major drop of force due to FZ failure at approximately the same displacement (24.0 mm) as in the experiments (experimental average: 23.7 mm). Notably, the actual crack initiation at a 22.5-mm displacement causes only a minor drop of force. Whereas the initiation takes place in the FZ adjacent to Z1 of the girder, the major drop of force is caused by a complete “cut” through the FZ adjacent to Z1 of the struts (Table 3, 24.1-mm column). Thus, the numerical model reproduces well the experimentally observed results (Figure 20). Table 3 also shows the evolution of the η- and $\varepsilon_{eq,pl}$-distribution at the appropriate displacements. It is visible that cracks initiate in the FZ at a stress triaxiality just above zero and at a strain of about 15%. With increasing displacement, more and more areas within the FZ reach and exceed η ≈ 0, and therefore damage starts to accumulate faster. Thus, at a 41-mm displacement, the whole remaining cross section of the FZ fails. In addition, crack initiation in Z1 occurs adjacent to Z2 in the girder on the surface at η > 1/3. The combination of crack initiation in Z1 and complete failure of the FZ results in the second drop of force in the simulations (beginning at a 36-mm displacement). The location and growth direction of the cracks within the simulations with the RTCL criterion are the same as for the BW criterion, but due to less damage accumulation in the FZ, both major drops of force occur at higher displacements and compression forces.

**Figure 23:**
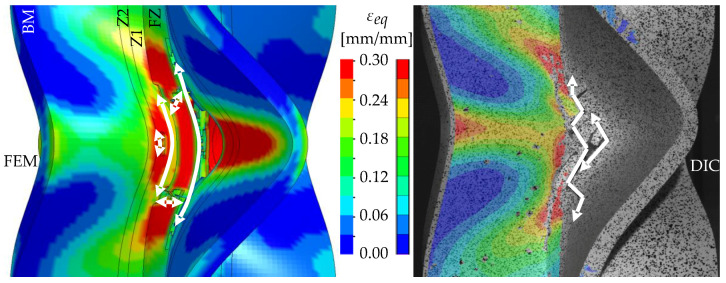
Comparison of crack growth in the FE model (BW criterion) and experiment at 45-mm displacement.

Figure 23 shows that the strain distribution in the simulations is in good agreement with the experimental results. Furthermore, the crack propagation in the FZ in the axial direction is also reproduced well in the simulation by deleting elements. In the HAZ of the girder, crack initiation occurs in Z1 adjacent to Z2 in the simulations (Table 3, 41-mm column), compared to the experimentally observed crack initiation directly adjacent to the FZ in the center of the girder. Due to the discontinuous material properties, the stress triaxiality at the transition from Z2 to Z1 is increased. Furthermore, as a result of the calibration procedure in Z1, less damage is accumulated at η≈0 (i.e., in the center of the girder) than at η > 1/3 (i.e., at the transition from Z1 to Z2). This combination leads to the simulated crack initiation at a distance to the FZ at η > 1/3. However, the reduction of load-bearing capacity is mainly caused by the length of the cracks in the FZ and the subsequent reduction of membrane stiffness of the plate. Accordingly, the difference of the location of crack initiation in the HAZ compared to the experiments has only a minor influence on the load-bearing capacity, because the global length and direction of the cracks is modeled in good agreement with the experiments.

All results show that crack initiation in the FZ has the largest effect on the load-bearing capacity at displacements higher than 20 mm. This is in accordance with the literature [1], since fracture of the FZ reduces the membrane stiffness of the plates substantially. Moreover, the numerical results for η at crack initiation as well as the local crack growth direction in the experiments indicate shear-dominated crack initiation within the FZ. Consequently, $\varepsilon_{f,s}$ is expected to have the highest influence on the numerical results with the BW criterion. An additional simulation with an increased value of $\varepsilon_{f,s}$ = 0.10 shows later crack initiation (at a displacement of 24.9 mm), thus supporting this assumption. Furthermore, the calibration of the fracture criteria explains why the FZ fractures first, despite the HAZ having the lowest strength. With fracture strains of $\varepsilon_{f,s}$ = 0.08 and $\varepsilon_{f,ut}$ = 0.10, the FZ is less ductile than the HAZ ($\varepsilon_{f,s}$ = 2.20 and $\varepsilon_{f,ut}$ = 0.44). While in the smooth tensile tests perpendicular to the weld, the HAZ deforms almost unconstrained and therefore exhibits a large strain localization until fracture, no such localization is possible in the axial compression tests. In fact, at damaging stress triaxialities (i.e., for η > −1/3), the FZ is strained more than the HAZ. The low ductility of the FZ itself can be explained by the pores within the weld material (Figure 21), which are present in all welds investigated in this study.

## 4. Conclusions

The aim of this study was to investigate the fracture behavior of welded aluminum plates under axial compression. Therefore, two fracture criteria (RTCL and BW) have been calibrated for the base material, heat-affected zone and fusion zone in MIG-welded aluminum specimens. The calibration was conducted on tensile and shear tests, which were simulated numerically. The experimental results show that these material zones behave quite differently. Whereas the BM has the highest strength, the welding process leads to the lowest strength within in HAZ. However, especially the shear tests show that the ductility can increase in the HAZ. Additionally, the fracture path in shear tests in the FZ is different from the paths in the BM and HAZ. From the numerical results, the following conclusions can be drawn:

- The BW and RTCL criteria behave similarly in tension and are able to model the fracture behavior of all material zones in accordance with the experiments except for very high stress triaxialities in the BM.
- The BW criterion is more accurate than the RTCL under shear loading due to the second calibration parameter.
- The simulated crack path in the present shear tests is dependent on the combination of both BW parameters due to the inhomogeneity of the stress state.
- The approximation of the material inhomogeneity in the HAZ with two zones is sufficient to model the global behavior of tension specimens perpendicular to the weld. However, this subdivision leads to an underestimation of the local fracture strains in tension tests compared to the experiments. Subsequently, in shear tests the global fracture displacement was underestimated.

Furthermore, axial compression tests on welded X-profiles were conducted. The experimental results show the following:

- The global and local behavior is similar for all specimens and plate thicknesses;
- Crack initiation can occur in the FZ despite the lowest strength being found in the HAZ;
- Crack initiation does not necessarily occur in the location with the highest strain;
- Crack initiation occurs on higher displacements, when the plate thickness is increased due to later stability failure and subsequently later strain localization;
- Visible cracks do not affect the load-bearing capacity of the structure before a certain amount of crack coalescence occurs;
- Up to a local strain rate of 1/s, no strain rate effects are visible.

The axial compression tests have been simulated numerically with the two fracture criteria. The experimental results have been used to validate the numerical simulations. With the chosen approach, it is possible to simulate the global behavior of the X-profiles as a result of the local fracture behavior in good agreement with the experiments, when considering the BW criterion. The results show that calibrations on shear specimens improve the accuracy of the simulations, as the crack initiation occurs at low stress triaxiality within the FZ. Furthermore, modifying the shear-dominating parameter of the BW criterion in the FZ shows the strong influence of the ductility of the FZ on the global response of the welded X-profiles.

The ultimate strength prediction methods available for steel-stiffened panels cannot be directly applied to aluminum structures, partly because of the softening effects in the heat-affected zones near fusion weld lines. With the improved understanding of local failure mechanisms and the appropriate finite element modeling of welded joints the load bearing capacity of aluminum ship constructions can be estimated with more accuracy, enabling a better risk assessment during the ship design.

In future work, the underestimation of the fracture strain in the HAZ in tension can be addressed by subdividing the HAZ into more zones, thus reducing the present averaging effect in Z1. Therefore, additional thermomechanical simulations and analyses, extrapolations from local DIC results or experiments have to be conducted to obtain the necessary stress–strain and fracture behavior. With the use of a coupled damage model, damage-induced softening in shear tests of the BM can be considered in the simulations. However, the numerical simulation of the coalescence of microcracks initiating on pores under shear load is still a subject of current research.

## Figures and Tables

**Table 1:** Calibrated parameters for the RTCL and BW criterion.

	RTCL	BW	
	$\varepsilon_{f,ut}$	$\varepsilon_{f,ut}$	$\varepsilon_{f,s}$
**BM**	0.81	0.94	0.78
**Z2**	0.59	0.69	1.49
**Z1**	0.36	0.43	2.20
**FZ**	0.10	0.10	0.08

**Table 2:** Deformation of X-profiles (10-mm plate thickness) and crack growth within axial compression tests at different global displacements (data from [27]).

Displ.	X02	X04	X05
25 mm Cracks traced with white lines	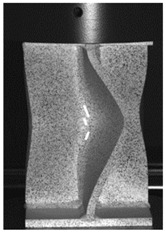	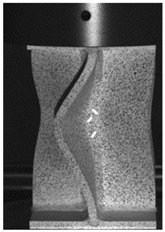	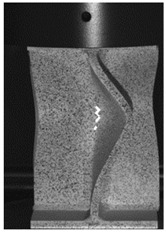
42 mm	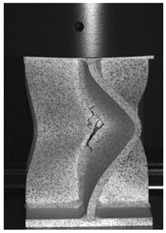	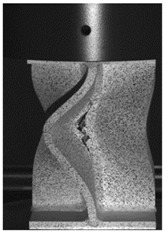	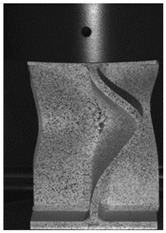

**Table 3:** Crack initiation and growth in the weld area simulated with the calibrated BW criterion.

	22.5 mm	24.1 mm	41.0 mm
**BM** **Z2** **Z1** **FZ**